# *MKI67* Enrichment Characterizes a Proliferative and Genomically Unstable Phenotype in Pancreatic Ductal Adenocarcinoma

**DOI:** 10.3390/cancers18162631

**Published:** 2026-08-14

**Authors:** Caleb Nathaniel Seavey, Kei Kawashima, Asama Khan, Élise Di Lena, Kazuaki Takabe, Mashaal Dhir

**Affiliations:** 1 Department of Surgical Oncology, Roswell Park Comprehensive Cancer Center, Buffalo, NY 14203, USA; 2 Department of Gastroenterological Surgery, Yokohama City University Graduate School of Medicine, Yokohama 236-0004, Japan; 3 Division of Surgical Oncology, Department of Surgery, Virginia Commonwealth University School of Medicine and Massey Cancer Center, Richmond, VA 980645, USA; 4 Department of Surgery, University at Buffalo Jacob School of Medicine and Biomedical Sciences, The State University of New York, Buffalo, NY 14203, USA; 5 Department of Surgery, Niigata University Graduate School of Medical and Dental Sciences, Niigata 951-8520, Japan; 6 Department of Breast Surgery and Oncology, Tokyo Medical University, Tokyo 160-8402, Japan

**Keywords:** pancreatic adenocarcinoma, Ki67, *MKI67*

## Abstract

Background/Objectives: Ki67 is a well-established histologic proliferation marker across many cancers; however, its clinical application in pancreatic ductal adenocarcinoma (PDAC) has remained limited. Multiple studies have demonstrated that Ki67 is prognostic in PDAC, with higher Ki67 expression associated with worsened outcomes. In this study, we aimed to characterize the underlying biology of Ki67-enriched tumors. Methods: Clinical and transcriptional data from PDAC patients were obtained from The Cancer Genome Atlas (TCGA) and three Gene Expression Omnibus (GEO) datasets. Mutational analysis from the TCGA cohort was also performed. Patients within each study were stratified into low- and high-expression groups based on the median transcript levels. Each dataset was analyzed independently, and differences between groups were subsequently compared across studies. Results: Consistent with their enhanced proliferative capacity, *MKI67*-high tumors were consistently enriched in pro-proliferative cellular pathways including E2F targets, MYC targets, and mitotic spindle formation as well as pro-growth metabolic pathways across all studies. *MKI67*-high PDAC is associated with higher histologic grading with similar pathologic staging. High expression was associated with worsened overall and disease-free survival in the TCGA cohort, although this was not consistently observed across external validation datasets. MKI67-high tumors exhibited a significantly higher mutational burden than the *MKI67*-low tumors. Conclusions: *MKI67* enrichment in PDAC correlates with aggressive tumor biology, enhanced proliferative signaling, genomic instability, and distinct microenvironmental and immune features. *MKI67*-high tumors demonstrate a reproducible association with proliferative signaling, genomic instability, and aggressive biological features across multiple transcriptomic datasets. Further validation is required to determine the clinical utility of *MKI67* as a biomarker in PDAC.

## Simple Summary

Pancreatic ductal adenocarcinoma (PDAC) is a biologically aggressive cancer, yet outcomes vary widely between patients. Ki67, encoded by the MKI67 gene, is a marker of cellular prolifration and is used across many cancers, but not routinely in PDAC. Using transcriptomic and clinical data from the TCGA cohort and three independent GEO datasets, this study characterized the biology of PDAC with high MKI67 expression. MKI67-high tumors were clinically associated higher histologic grade despite similar pathologic stage. These tumors were consistently enriched in proliferative pathways (E2F and MYC targets, mitotic spindle formation) and pro-growth metabolic programs. They also showed greater genomic instability, higher mutational burden, more frequent KRAS and TP53 mutations, and a distinct immune and stromal microenvironment. Although high expression predicted worse survival in TCGA, this was not reproduced across validation cohorts. Overall, MKI67 enrichment identifies a proliferative, genomically unstable, biologically aggressive PDAC phenotype, warranting further validation as a prognostic biomarker.

## 1. Introduction

Pancreatic ductal adenocarcinoma (PDAC) remains the third most common cause of cancer-related mortality in the United States [1,2]. Even among patients with localized disease who undergo margin-negative resection in conjunction with multi-agent chemotherapy, long-term outcomes remain poor, thus highlighting the overall aggressive nature of PDAC [1,2,3,4]. While the overall prognosis remains poor, PDAC is still associated with marked heterogeneity in clinical behavior [4,5,6]. Notably, among patients with localized disease, some experience prolonged disease-free survival following surgical resection, whereas others develop rapid recurrence and early mortality despite similar treatment paradigms [3].

Given the substantial morbidity associated with pancreatic resection, patients who recur early after surgery are considered to have yielded limited benefit from local therapy [7,8]. Accordingly, understanding the biology of these aggressive-type tumors may aid in prognostication and may elucidate unique and potentially targetable mechanisms of tumorigenesis and progression. Further, biomarkers that distinguish biologically aggressive tumors from more indolent tumors may inform patient selection and optimize sequencing of systemic therapy and surgery.

Ki67 is a nuclear protein that is expressed during the active phases of the cell cycle (G1, S, G2, M) but is absent in resting cells [9,10]. The regulation of Ki67 protein expression is primarily governed by mRNA transcription of the *MKI67* gene and has limited post-transcriptional and post-translational regulation effects on protein abundance [9]. Nuclear expression of this protein has been validated and is clinically used in multiple other malignancies as a marker of proliferative activity [11,12,13]. Previous reports in PDAC have shown that the Ki67 index can predict overall, disease-free, and disease-specific survival and correlates with histologic grading [14,15]. However, at present, Ki67 immunohistochemistry is not routinely performed for PDAC.

Despite the prognostic association of Ki67 with survival, little is known about the biology of Ki67-high tumors. Previous work has demonstrated the utility of *MKI67* transcriptional activity as a marker for Ki67 abundance, as the protein expression control of Ki67 is determined by *MKI67* mRNA regulation [16,17]. Herein, we aimed to investigate the molecular, genomic, and tumor microenvironmental features associated with *MKI67*-high PDAC and evaluate its potential utility as an indicator of biologically aggressive disease in patients with localized pancreatic cancer.

## 2. Materials and Methods

### 2.1. Clinical and Transcriptomic Data Acquisition

We utilized The Cancer Genome Atlas (TCGA) pancreatic adenocarcinoma (PAAD) cohort that consists of primary pancreatic ductal adenocarcinoma (PDAC) patients who underwent surgical resection and had available transcriptomic data, similarly to our previous studies [18,19,20,21,22]. Clinicopathologic parameters were similarly extracted from the dataset through the Genomic Data Commons Data Portal through cBioportal and using the TCGAbiolinks package (version 2.36.0) in R. Perineural invasion (PNI) status was obtained from the pathology dataset generated by Kefeli & Tatonetti 2024 [23].

External validation was performed using separate cohorts from the Gene Expression Omnibus (GEO) database with available normalized gene expression data and overall survival. Datasets from GEO included GSE85916 (GEO1), GSE71729 (GEO2), and GSE21501 (GEO3). GSE85916 (GEO1) included 80 samples of curative-intent resected pancreatic adenocarcinoma which underwent transcriptional profiling. GSE71729 (GEO2) included 145 samples of primary PDAC tumors which underwent transcriptional analysis [6]. GSE21501 (GEO3) included 132 samples of resected PDAC which underwent transcriptional profiling [24].

Patients in each dataset were divided into two groups based on median *MKI67* gene expression. Those with *MKI67* expression greater than the median value within each cohort were designated as the highly proliferative group (*MKI67*-high), while the remaining patients were categorized as *MKI67*-low. This within-cohort median stratification was chosen to minimize platform-dependent variability across RNA sequencing and microarray datasets and minimize platform-related bias. As no pooled transcriptomic analysis across the multiple studies was performed, batch correction across datasets was not required.

All transcriptomic analyses were conducted using log2-transformed normalized transcriptomic data. The TCGA and GEO datasets utilized in this study are publicly available and deidentified; therefore, Institutional Review Board approval was waived.

### 2.2. Gene Set Enrichment Analysis (GSEA)

Gene set enrichment analysis (GSEA) was performed using ranked gene lists derived from differential gene expression analysis between *MKI67*-high and *MKI67*-low tumors. Differentially expressed genes were identified using the *limma* package (version 3.63.3) in R. GSEA was then performed using the *fgsea* package (version 1.34.2) in a pre-ranked manner based on log2 fold-change values. The Hallmark gene set from the Molecular Signatures Database (MSigDB) was used as a reference gene set similarly to our previous studies [25,26,27,28].

Statistical significance for pathway enrichment was defined by a false discovery rate (FDR) < 0.25. FDR values were calculated based on the Benjamini–Hochberg method integrated within the *fgsea* package. Normalized enrichment scores (NESs) were calculated for each pathway, and enrichment results were compared across independent datasets to evaluate reproducibility.

### 2.3. Tumor Microenvironment and Mutational Analysis

The xCell algorithm was used for immune cell and stromal cell composition analysis using the xCell package (version 1.1.0) in R, as previously described. Differences in cell composition were considered significant if two of the four studies found similar significant results. Scored mutational analysis and immune phenotypes of the PAAD TCGA dataset were obtained as previously published by Thorsson et al., 2018 [29]. Immune and stromal cell composition analyses were considered exploratory; therefore, unadjusted *p*-values are reported, with emphasis placed on findings that were consistently observed across multiple independent cohorts rather than on statistical significance within any single dataset.

### 2.4. Statistical Analysis

Statistical significance for comparisons between *MKI67*-high and *MKI67*-low groups was defined as *p* < 0.05. Continuous variables were compared using the Mann–Whitney U test or Kruskal–Wallis test as appropriate. Categorical variables were analyzed using two-tailed Fisher’s exact tests or chi-square tests. Survival analyses were performed using the Kaplan–Meier method and compared using the log-rank test. Univariable and multivariable Cox regression analysis was performed on the TCGA cohort. Overall survival and disease-free survival were analyzed when available. Cell deconvolution analysis was considered exploratory so multiple-comparison adjustment was not performed.

All statistical analyses and data visualization were performed using R (version 4.5.1) with Bioconductor (version 3.21). All tests were two-sided.

## 3. Results

### 3.1. MKI67-High PDAC Is Associated with Worse Survival in the TCGA but Not the GEO Cohorts

**Figure 1:**
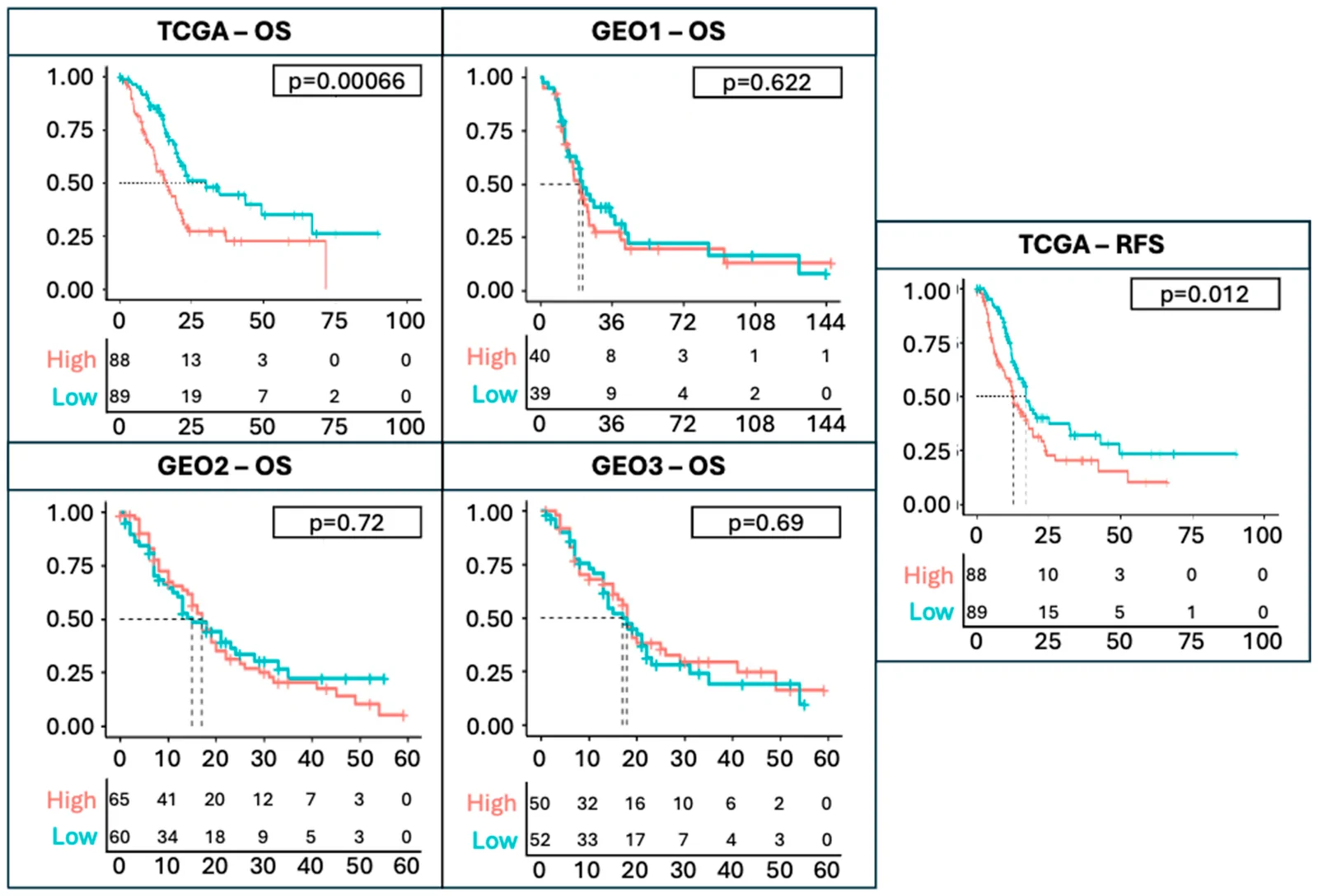
Survival analysis for overall and recurrence-free survival across studies. Kaplan–Meier curves comparing *MKI67*-high tumors (red) vs. *MKI67*-low tumors (blue). Numbers below signify number at risk. *X*-axis shows time interval in months. Dotted lines represent median survival. OS—Overall Survival, RFS—Recurrence-Free Survival.

We evaluated the prognostic significance of *MKI67* transcriptional expression in PDAC. In the TCGA cohort, *MKI67*-high tumors were associated with significantly worse disease-free survival (*p* = 0.012) and overall survival (*p* = 0.00066) compared with *MKI67*-low tumors (Figure 1). In contrast, no statistically significant difference in overall survival was observed between *MKI67*-high and *MKI67*-low groups in the three independent GEO cohorts (Figure 1). These findings suggest that while *MKI67* enrichment may identify biologically aggressive tumors, its prognostic significance may be influenced by cohort-specific factors including treatment heterogeneity, patient selection, and transcriptomic platform differences.

On univariable Cox proportional hazard ratio analysis of overall survival, increased age, greater T stage, nodal positivity and *MKI67*-high tumors are associated with worsened overall survival (Supplementary Table S1). Further, age, nodal positivity, and *MKI67*-high tumors remain statistically significantly associated with worsened overall survival on multivariable analysis (Supplementary Table S2).

### 3.2. MKI67-High Tumors Correlate with Higher Histologic Grade Independent of Pathologic Stage and an Aggressive Phenotype

**Figure 2:**
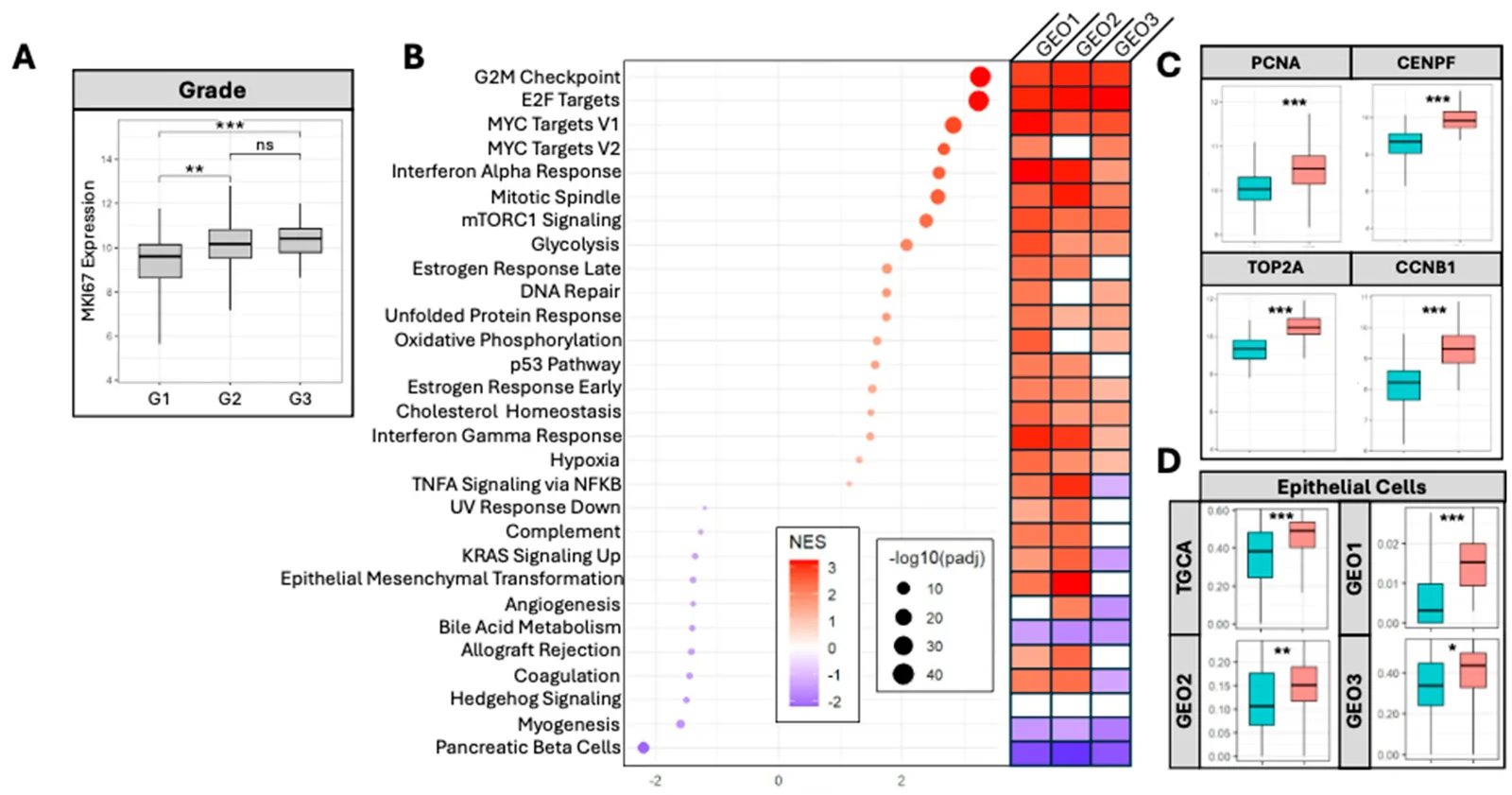
*MKI-67* tumors are associated with a proliferative phenotype. (**A**) Box plot of *MKI67* expression based on histologic grade (G1, G2, G3). Pairwise *p* values from a Mann–Whitney test shown above. ns: nonsignificant, ** *p* < 0.01, *** *p* < 0.001. (**B**) (Left) Bubble plot demonstrating Hallmark gene set enrichment analysis (GSEA) comparing *MKI67*-high and *MKI67*-low tumors. Bubble size corresponds to statistical significance (adjusted *p*-value/FDR) and color corresponds to normalized enrichment score (NES). A positive NES indicates enrichment in *MKI67*-high tumors in the TCGA dataset. NES: normalized enrichment score, padj: adjusted *p* value/false discovery rate. (Right) Heatmap of normalized enrichment scores from GSEA of the identified differentially expressed gene sets in the GEO validation cohorts. Non-differentially expressed gene sets were censored. (**C**) Box plot of canonical proliferative marker expression level. Blue: *MKI67*-low, red: *MKI67*-high. *** *p* < 0.001. (**D**) Box plots of epithelial cell component from xCell. Blue: *MKI67*-low, red: *MKI67*-high; significance was calculated with a Mann–Whitney U test., * *p* < 0.05, ** *p* < 0.01, *** *p* < 0.001.

Clinicopathologic characteristics were compared within the TCGA cohort to determine whether *MKI67* expression was associated with differences in traditional staging parameters. *MKI67*-high and *MKI67*-low groups demonstrated no significant differences in age, sex, race, T stage, nodal status, AJCC stage, or margin status (Table 1). However, *MKI67*-high tumors were significantly associated with higher histologic grade compared with *MKI67*-low tumors (*p* = 0.004) (Table 1). Specifically, grade 1 tumors are associated with significantly lower *MKI67* transcriptional levels when compared to higher grade tumors (G1 vs. G2 *p* = 0.003, G1 vs. G3 *p* = 0.001) with no significant difference between grade 2 and grade 3 tumors (*p* = 0.30) (Figure 2A).

To characterize the biological pathways associated with elevated proliferative activity, we performed gene set enrichment analysis (GSEA) comparing *MKI67*-high and *MKI67*-low tumors using the Hallmark gene sets. Across the cohorts, *MKI67*-high tumors demonstrated enrichment of canonical cell-cycle and proliferation-related pathways, including E2F targets, G2M checkpoint, MYC targets, and mitotic spindle assembly. These findings validate *MKI67* transcriptional expression as a marker of increased proliferative activity in PDAC (Figure 2B). As well, *MKI67*-high tumors coexpress other canonical proliferative markers such as PCNA, CENPF, TOP2A, and CCNB1 (Figure 2C). Finally, on cell composition analysis, *MKI67*-high tumors demonstrated enrichment of epithelial cell subtypes, likely reflecting increased tumor cell purity (Figure 2D).

### 3.3. MKI67-High Tumors Are Enriched in Metabolic Programs Without Enrichment in Canonical PDAC-Altered Pathways

In addition to cell-cycle signaling enrichment, *MKI67*-high tumors were consistently enriched in metabolic pathways necessary to sustain rapid cellular division, including glycolysis, oxidative phosphorylation, cholesterol homeostasis, and hypoxia signaling. In addition, *MKI67*-high tumors were associated with enrichment in genomic repair mechanisms, notably p53 signaling and DNA repair, which may support ongoing cell proliferation despite genomic stress. Finally, *MKI67*-enriched tumors were enriched in interferon signaling (alpha and gamma) (Figure 2B).

Notably, several signaling pathways traditionally implicated in PDAC pathogenesis, Hedgehog signaling, WNT/β-catenin signaling, and epithelial-to-mesenchymal transition (EMT), were not enriched in *MKI67*-high tumors [30,31,32,33,34]. This finding suggests that the highly proliferative phenotype associated with *MKI67* overexpression is not secondarily associated with derangements in these canonical developmental or EMT-associated pathways. Of note, mTOR signaling, which is known to drive PDAC pathogenesis, was significantly enriched in *MKI67*-high tumors (Figure 2B) [35].

### 3.4. MKI67-High Tumors Exhibit Greater Genomic Instability and Mutational Burden

**Figure 3:**
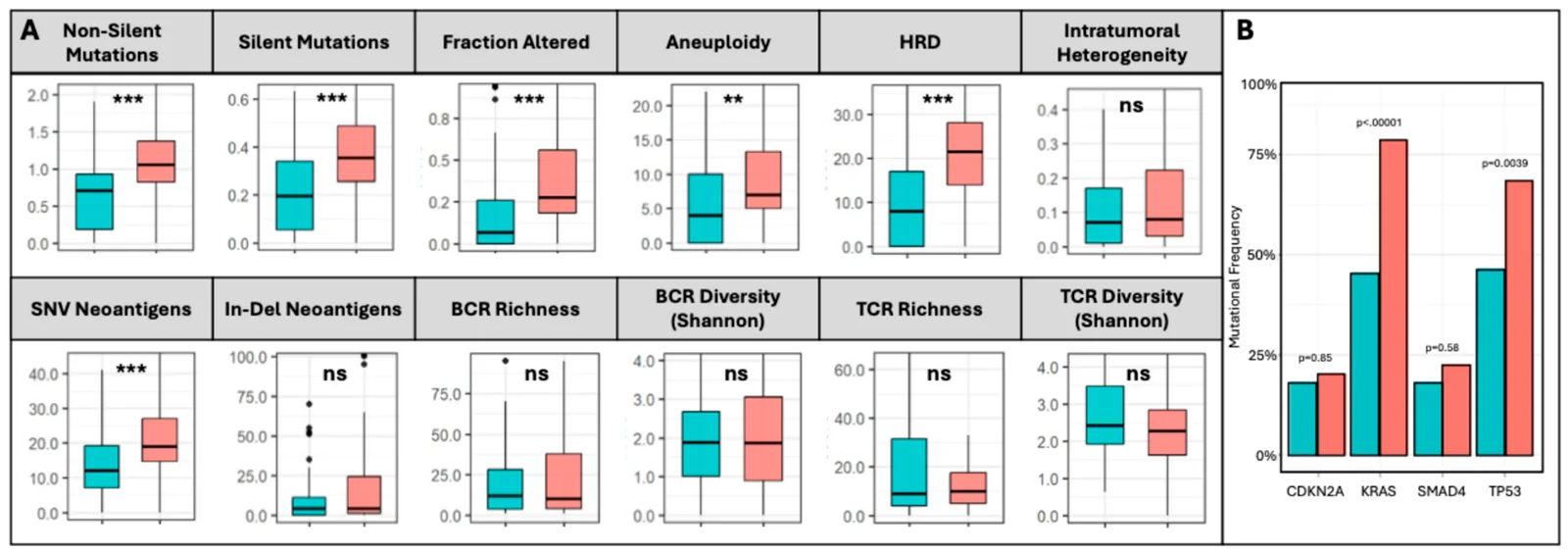
Mutation analysis and immune response signatures of MKI67-high vs. -low tumor. (**A**) (**Top**) Box plots of mutational signatures from Thorsson et al. [29] HRD: homologous recombination deficiency. (**Bottom**) Box plots of neoantigen diversity and T cell and B cell response signatures from Thorsson et al. [29] Blue: *MKI67*-low, red: *MKI67*-high; significance was calculated with a Mann–Whitney U test. ns: nonsignificant, ** *p* < 0.01, *** *p* < 0.001. (**B**) Bar graph of mutational frequencies of canonical PDAC-associated genes (*CDKN2A*, *KRAS*, *SMAD4*, *TP53*). Significance was calculated with a chi-squared test with *p* values displayed above the bars. Blue: *MKI67*-low, red: *MKI67*-high.

Since cancer with high mutation burden is often highly proliferative, we next examined the mutational landscape of *MKI67*-high tumors within the TCGA dataset. *MKI67*-high tumors demonstrated a significantly greater burden of both non-silent and silent mutations, as well as a larger fraction of the overall genome altered (Figure 3A). In terms of copy number variations, *MKI67*-high tumors exhibited increased aneuploidy and greater homologous recombination deficiency (HRD) scores. There was a similar degree of intratumoral heterogeneity between groups (Figure 3A).

Regarding genes that are consistently mutated in PDAC, *MKI67*-high tumors demonstrated increased mutational frequency of KRAS and TP53, whereas alterations in CDKN2A and SMAD4 occurred at similar rates between groups (Figure 3B). Collectively, these findings suggest that *MKI67*-high tumors possess a genomically unstable and mutation-enriched phenotype.

Cancers with a higher mutational burden have a greater diversity of neoantigens. Therefore, we analyzed if there was a differential abundance of neoantigens and whether there were resultant differences in T and B cell receptor repertoires. We identified a significant increase in single-nucleotide variant (SNV)-driven neoantigens with a similar abundance of in-del neoantigens. However, both B cell (BCR) and T cell receptor (TCR) richness and diversity were similar between groups (Figure 3A).

### 3.5. MKI-High Tumors Display a Unique Immune Tumoral Microenvironment

**Figure 4:**
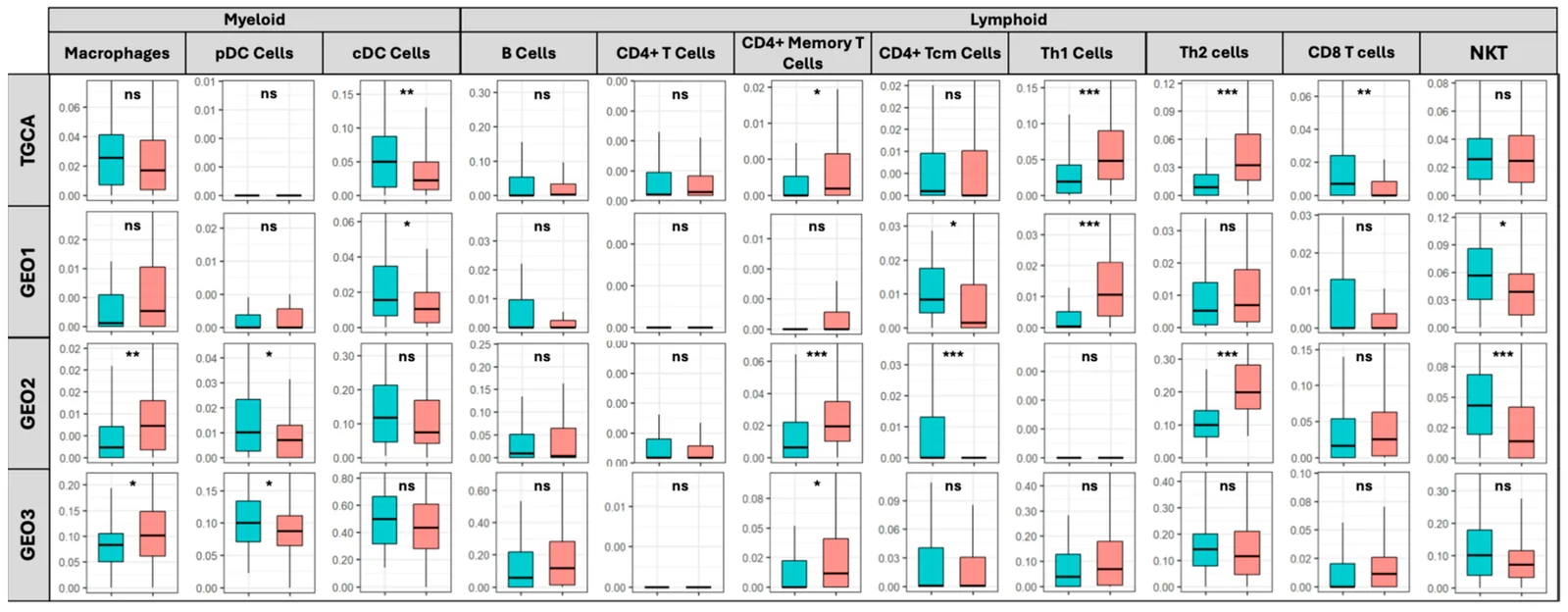
Immune cell composition analysis of MKI67-high vs. low-tumors. Box plots of antitumor cell and immune cell composition from xCell (macrophages; pDC: plasmocytic dendritic cells; cDC: conventional dendritic cells, B cells, CD4+ T cells, CD4+ memory T cells; CD4+ Tcm: CD4+ central memory T cells; Th1: T helper 1 cells; Th2: T helper 2 cells, CD8+ T cells; NKT: natural killer T cells). Cellular composition was estimated using the xCell transcriptomic deconvolution algorithm. Results represent inferred relative enrichment scores rather than directly measured cell abundance. Blue: *MKI67*-low, red: *MKI67*-high; significance was calculated with a Mann–Whitney U test. ns: nonsignificant, * *p* < 0.05, ** *p* < 0.01, *** *p* < 0.001.

First, we estimated differences in immune cell composition between *MKI67*-high and *MKI67*-low tumors using the xCell computational deconvolution algorithm. MKI67-high tumors were enriched in Th1 and Th2 T helper subtypes as well as CD4+ memory T cells and macrophages. In comparison, *MKI67*-low tumors were enriched in both plasmacytoid and conventional dendritic cell subtypes, central memory T cells, and NKT cells (Figure 4). The proportions of tumor-resident immune cell populations within broader immune cell subtypes were similarly distributed between groups on EPIC cell deconvolution (Supplementary Figure S1).

### 3.6. MKI67 Enrichment Is Associated with Changes in the Nonimmune Cell Composition

**Figure 5:**
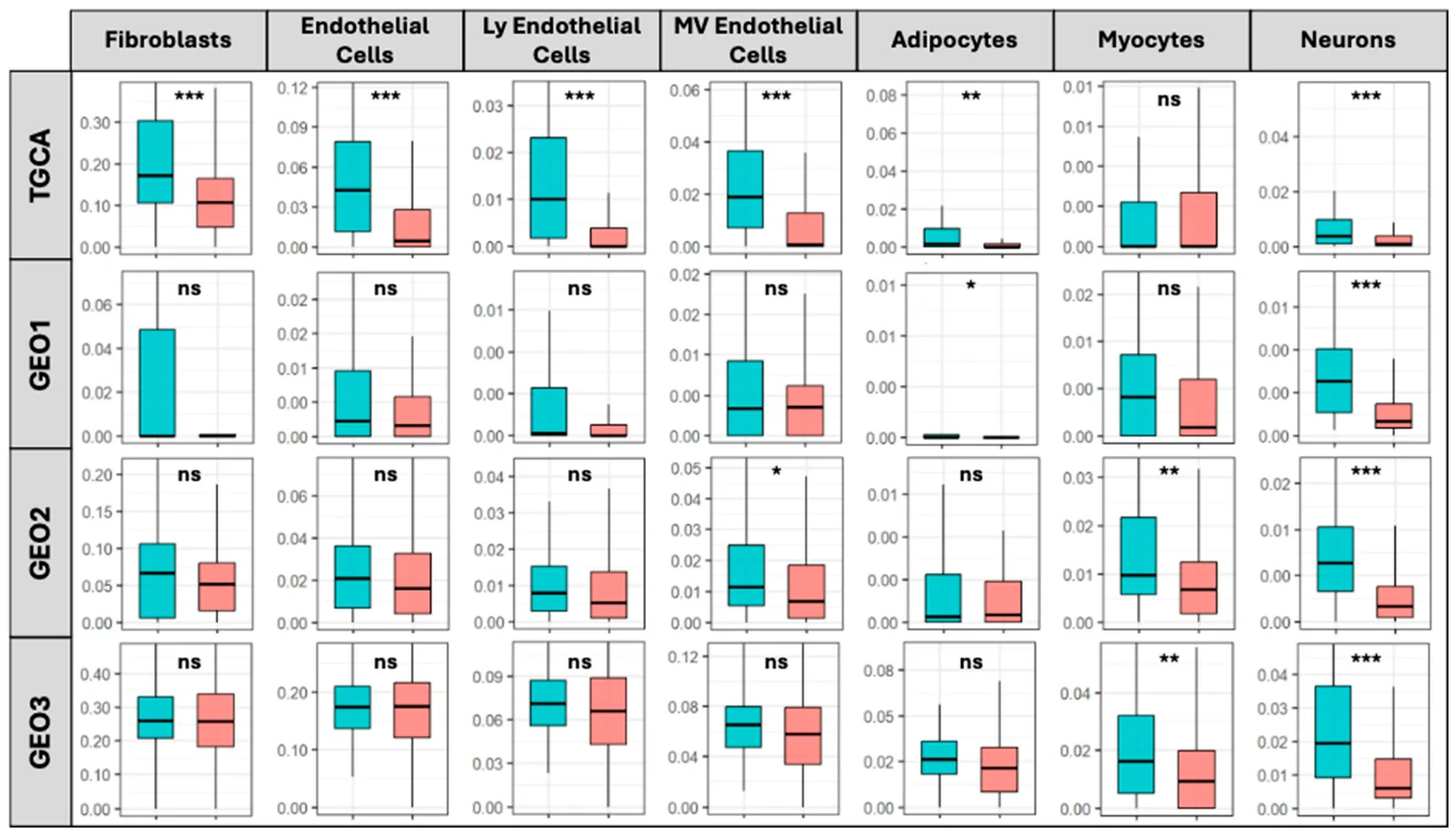
Nonimmune cell composition analysis of *MKI67*-high vs. -low tumors. Box plots of stromal cell composition from xCell (fibroblasts, endothelial cells; Ly: lymphatic endothelial cells; MV: microvascular endothelial cells, adipocytes, myocytes, and neurons). Cellular composition was estimated using the xCell transcriptomic deconvolution algorithm. Results represent inferred relative enrichment scores rather than directly measured cell abundance. Blue: *MKI67*-low, red: *MKI67*-high; significance was calculated with a Mann–Whitney U test. ns: nonsignificant, * *p* < 0.05, ** *p* < 0.01, *** *p* < 0.001.

In terms of stromal cell composition, *MKI67*-low tumors exhibited higher proportions of microvascular endothelial cells, adipocytes, and myocytes. Interestingly, *MKI67*-low tumors also demonstrated higher neuronal cell enrichment, although rates of histologic perineural invasion were similar between groups (Figure 5, Table 1). On EPIC cell deconvolution, MKI67-high tumors exhibited reduced enrichment of endothelial cell populations compared with MKI67-low tumors.

## 4. Discussion

In this study, we demonstrate that *MKI67* enrichment is associated with a biologically aggressive phenotype of pancreatic ductal adenocarcinoma characterized by enhanced proliferative capacity, a unique genomic phenotype, and a tumor microenvironment distinct from the *MKI67*-low cohort. Collectively, these findings support *MKI67* as a biologically relevant marker that predicts tumor aggressiveness beyond conventional clinicopathologic staging.

As expected, at the transcriptomic level, *MKI67*-high tumors were enriched for canonical cell-cycle and proliferation-associated pathways, including E2F targets, G2M checkpoint regulation, MYC signaling, and mitotic spindle formation. This is particularly relevant as previous work using the TCGA demonstrated that the top signaling pathways enriched in tumors with early recurrence in PDAC were these same pathways [36,37]. The proliferative transcriptional program observed in *MKI67*-high tumors shares features with previously described molecular subtypes of PDAC. Prior transcriptomic studies have identified a “basal-like” or “squamous” subtype characterized by enhanced MYC signaling, cell-cycle activation, and poor clinical outcomes [38]. Many of the pathways enriched in the *MKI67*-high tumors in the present study, including E2F targets, G2M checkpoint regulation, and MYC signaling, overlap with signatures reported in this aggressive PDAC subtype. While formal subtype classification was not performed in the current analysis, these findings suggest that *MKI67* transcriptional enrichment may capture a proliferative phenotype that partially overlaps with previously described basal-like biology. Further studies integrating *MKI67* expression with established PDAC subtype classifiers may help clarify this relationship.

In addition, enrichment of metabolic pathways such as glycolysis, oxidative phosphorylation, cholesterol homeostasis, and hypoxia underscores the metabolic adaptations required to sustain rapid proliferation. Interestingly, several signaling pathways traditionally implicated in PDAC development and progression, notably Hedgehog signaling, WNT/β-catenin signaling, and epithelial-to-mesenchymal transition, were less enriched in *MKI67*-high tumors, suggesting that the proliferative capacity associated with these tumors may be driving aggressive phenotypes in a unique fashion distinct from these well-described pathways [30,31,32,33,34].

Clinically, *MKI67*-high tumors were associated with inferior overall and disease-free survival in the TCGA cohort despite similar clinical staging and margin status. However, these survival associations were not reproduced across the external validation cohorts. Several factors may explain this discrepancy, including differences in patient selection, treatment strategies, sample size, transcriptomic platforms, and available clinical annotation. Therefore, while *MKI67* enrichment consistently identified tumors with aggressive biological features, its prognostic value remains uncertain and warrants further validation in prospectively annotated cohorts.

Consistent with their aggressive behavior, *MKI67*-high tumors were associated with higher histologic grade with comparable AJCC staging. This finding reinforces the hypothesis that *MKI67* reflects intrinsic tumor aggressiveness rather than advanced anatomic extent. Histologic grade and percent of cells positive for Ki67+, however, can be subject to interobserver variability; *MKI67* may provide a complementary, quantitative biomarker to refine risk assessment [39,40].

Genomically, *MKI67*-high tumors exhibited a higher overall mutational burden, greater genomic instability, increased aneuploidy, and higher levels of homologous recombination deficiency. These features are consistent with rapid cellular turnover and impaired DNA damage repair. The increased frequency of *KRAS* and *TP53* mutations in *MKI67*-high tumors further supports a more aggressive oncogenic landscape.

In addition to genomic instability, *MKI67*-high tumors demonstrated features suggestive of increased immunologic activity, including higher single-nucleotide variant-derived neoantigen burden and enrichment of interferon signaling pathways, along with increased Th1 and Th2 immune signatures. These observations were derived from computational deconvolution methods and therefore represent inferred differences in relative cellular enrichment rather than direct measurements of immune cell abundance. Furthermore, the increased epithelial enrichment observed in *MKI67*-high tumors may contribute to some of the observed differences in immune and stromal signatures. Accordingly, these findings should be interpreted as hypothesis-generating and warrant validation using orthogonal methodologies such as immunohistochemistry, flow cytometry, or spatial transcriptomics.

Although pancreatic ductal adenocarcinoma is typically considered an immunologically “cold” tumor, these findings raise the possibility that highly proliferative tumors may exhibit relative immunogenic characteristics within this otherwise immune-resistant disease [41]. The coexistence of genomic instability and immune signaling suggests a complex tumor microenvironment in which increased antigenicity may be counterbalanced by immunosuppressive mechanisms. While these observations do not establish sensitivity to immunotherapy, and are based on computational estimates of cellular composition rather than direct measurement, they suggest that *MKI67*-high PDAC may possess a distinct immune contexture that warrants further investigation using orthogonal approaches such as spatial transcriptomics or immunohistochemical validation.

Although *MKI67* is traditionally viewed as a marker of cellular proliferation rather than a biological driver, emerging evidence suggests Ki67 may contribute to chromatin organization, heterochromatin maintenance, and cell-cycle progression. Nevertheless, the present study was not designed to determine causality, and the observed pathway enrichments should be interpreted as features associated with *MKI67*-high tumors rather than direct consequences of *MKI67* expression itself.

This study has several limitations. First, the analyses were retrospective and relied on publicly available transcriptomic datasets, which are subject to selection bias, variability in tissue processing, and heterogeneity in treatment regimens that could not be fully controlled for. Second, several clinically relevant variables, including neoadjuvant therapy, chemotherapy regimen, recurrence patterns, and molecular subtype classification, were incompletely available across datasets and therefore could not be comprehensively analyzed. Third, immune and stromal cell composition were inferred computationally using xCell and were not validated using orthogonal methodologies. Consequently, these analyses should be considered exploratory and require validation using orthogonal approaches such as immunohistochemistry or spatial transcriptomics. Fourth, *MKI67* expression was assessed at the transcriptomic level rather than by standardized immunohistochemistry. Finally, survival associations were not consistently reproduced across validation cohorts, highlighting the need for prospective validation studies before clinical implementation.

## 5. Conclusions

In summary, these findings suggest that *MKI67* transcriptional enrichment identifies a proliferative and genomically unstable subtype of PDAC characterized by aggressive biological features. From a translational perspective, *MKI67* expression may ultimately contribute to risk stratification strategies, although prospective validation will be required before clinical application. Prospective studies integrating *MKI67* assessment with clinical decision-making will be necessary to validate its utility as a biomarker in localized PDAC.

## Figures and Tables

**Table 1:** Clinicopathologic characteristics stratified by *MKI67* level in the TCGA cohort. Baseline demographics and pathologic staging of tumors in the TCGA dataset. *p*-values were calculated using Wilcoxon rank sum for continuous variables and Fisher’s exact or chi-square for categorical variables. SD: standard deviation.

	Overall	*MKI67*-High	*MKI67*-Low	*p*-Value
	*N* = 177	*N* = 89	*N* = 88	
**AGE (SD)**	64 (11)	65 (11)	64 (11)	0.4
**SEX**				
Female	80/177 (45%)	37/89 (42%)	43/88 (49%)	0.3
Male	97/177 (55%)	52/89 (58%)	45/88 (51%)	
**RACE**				
Asian	11/173 (6.4%)	7/89 (7.9%)	4/84 (4.8%)	0.2
Black/African American	6/173 (3.5%)	1/89 (1.1%)	5/84 (6.0%)	
White	156/173 (90%)	81/89 (91%)	75/84 (89%)	
Unknown	4	0	4	
**GRADE**				
G1	31/177 (18%)	8/89 (9.0%)	23/88 (26%)	0.004
G2	94/177 (53%)	47/89 (53%)	47/88 (53%)	
G3	48/177 (27%)	31/89 (35%)	17/88 (19%)	
G4	2/177 (1.1%)	1/89 (1.1%)	1/88 (1.1%)	
GX	2/177 (1.1%)	2/89 (2.2%)	0/88 (0%)	
**PERINEURAL INVASION**				
Positive	101/127 (80%)	51/59 (86%)	50/68 (74%)	0.072
Negative	26/127 (20%)	8/59 (14%)	18/68 (26%)	
Unknown	50	30	20	
**RADIATION TREATMENT**	43/160 (27%)	20/81 (25%)	23/79 (29%)	0.5
Unknown	17	8	9	
**pT STAGE**				
T1	7 (4.0%)	3 (3.4%)	4 (4.5%)	0.7
T2	23 (13%)	10 (11%)	13 (15%)	
T3	142 (81%)	74 (84%)	68 (77%)	
T4	3 (1.7%)	1 (1.1%)	2 (2.3%)	
TX	1 (0.6%)	0 (0%)	1 (1.1%)	
Unknown	1	1	0	
**pN STAGE**				
N0	49 (28%)	27 (30%)	22 (25%)	0.5
N1	119 (68%)	60 (67%)	59 (68%)	
N1B	4 (2.3%)	1 (1.1%)	3 (3.4%)	
NX	4 (2.3%)	1 (1.1%)	3 (3.4%)	
Unknown	1	0	1	
**PATHOLOGIC STAGE**				
STAGE I	1 (0.6%)	0 (0%)	1 (1.1%)	0.8
STAGE IA	5 (2.9%)	2 (2.3%)	3 (3.4%)	
STAGE IB	15 (8.6%)	6 (6.8%)	9 (10%)	
STAGE IIA	28 (16%)	17 (19%)	11 (13%)	
STAGE IIB	118 (67%)	59 (67%)	59 (68%)	
STAGE III	4 (2.3%)	2 (2.3%)	2 (2.3%)	
STAGE IV	4 (2.3%)	2 (2.3%)	2 (2.3%)	
Unknown	2	1	1	
**RESIDUAL DISEASE**				
R0	105 (63%)	49 (58%)	56 (68%)	0.3
R1	53 (32%)	32 (38%)	21 (26%)	
R2	5 (3.0%)	3 (3.5%)	2 (2.4%)	
RX	4 (2.4%)	1 (1.2%)	3 (3.7%)	
Unknown	10	4	6	

## Data Availability

All data presented in this article were derived from previously published, publicly available datasets.

## Supplementary Materials

The following supporting information can be downloaded at: https://www.mdpi.com/article/10.3390/cancers18162631/s1. Table S1: Univariable Cox proportional hazards analysis of overall survival in the TCGA Cohort. Univariable Cox proportional hazards regression analysis evaluating clinicopathologic factors associated with overall survival. Female sex, T1–2 disease, and N0 disease served as the reference categories for sex, T stage, and nodal status, respectively. Hazard ratios (HRs) are presented with 95% confidence intervals (CIs). Table S2: Multivariable Cox proportional hazards analysis of overall survival in the TCGA Cohort. Multivariable Cox proportional hazards regression analysis evaluating clinicopathologic factors associated with overall survival. Female sex, T1–2 disease, and N0 disease served as the reference categories for sex, T stage, and nodal status, respectively. MKI67 Hazard ratios (HRs) are presented with 95% confidence intervals (CIs). Figure S1: Immune and stromal cell composition analysis of MKI67-high vs. MKI67-low tumors. Box plots depicting inferred immune and stromal cell composition differences between *MKI67*-high and *MKI67*-low tumors using the EPIC cell deconvolution. Blue: MKI67-low tumors; Red: MKI67-high tumors. Statistical significance was assessed using the Mann–Whitney U test. *p* values are displayed above each comparison.

## Abbreviations

The following abbreviations are used in this manuscript:

PDAC	Pancreatic Ductal Adenocarcinoma
TCGA	The Cancer Genome Atlas
GEO	Gene Expression Omnibus
PNI	Perineural Invasion
MSigDB	Molecular Signatures Database
GSEA	Gene Set Enrichment Analysis
NES	Normalized Enrichment Score
SNV	Single-Nucleotide Variant
BCR	B Cell Receptor
TCR	T Cell Receptor
