# Supplementary material for: MKI67 Enrichment Characterizes a Proliferative and Genomically Unstable Phenotype in Pancreatic Ductal Adenocarcinoma
Source: Cancers (Basel). 2026 Aug 14;18(16):2631. doi: 10.3390/cancers18162631 (PMC13510446; doi:10.3390/cancers18162631)
Supplement: Supplementary file 1 [file cancers-18-02631-s001.zip › cancers-4431785-supplementary.pdf]

Table S1. Univariable Cox proportional hazards analysis of overall survival in the TCGA Cohort.

	**Hazard Ratio (95% CI)**	***p*-Value**
***MKI67* (High vs. Low)**	1.98 (1.29-3.04)	0.002
**Age**	1.03 (1.00-1.05)	0.023
**Sex (Male vs Female)**	0.75 (0.49-1.14)	0.178
**T stage (T3-4 vs T1-2)**	1.77 (0.91-3.43)	0.091
**N Stage (N1-2 vs N0)**	2.01 (1.19-3.37)	0.009

Univariable Cox proportional hazards regression analysis evaluating clinicopathologic factors associated with overall survival. Female sex, T1–2 disease, and N0 disease served as the reference categories for sex, T stage, and nodal status, respectively. Hazard ratios (HRs) are presented with 95% confidence intervals (CIs).

Table S2. Multivariable Cox proportional hazards analysis of overall survival in the TCGA Cohort.

	**Hazard Ratio (95% CI)**	***p*-Value**
***MKI67* (High vs. Low)**	2.11 (1.37–3.26)	<0.001
**Age**	1.02 (1.00–1.05)	0.028
**Sex (Male vs Female)**	0.74 (0.48–1.12)	0.157
**T stage (T3-4 vs T1-2)**	1.19 (0.59–2.37)	0.629
**N Stage (N1-2 vs N0)**	1.94 (1.12–3.36)	0.018

Multivariable Cox proportional hazards regression analysis evaluating clinicopathologic factors associated with overall survival. Female sex, T1–2 disease, and N0 disease served as the reference categories for sex, T stage, and nodal status, respectively. MKI67 Hazard ratios (HRs) are presented with 95% confidence intervals (CIs).

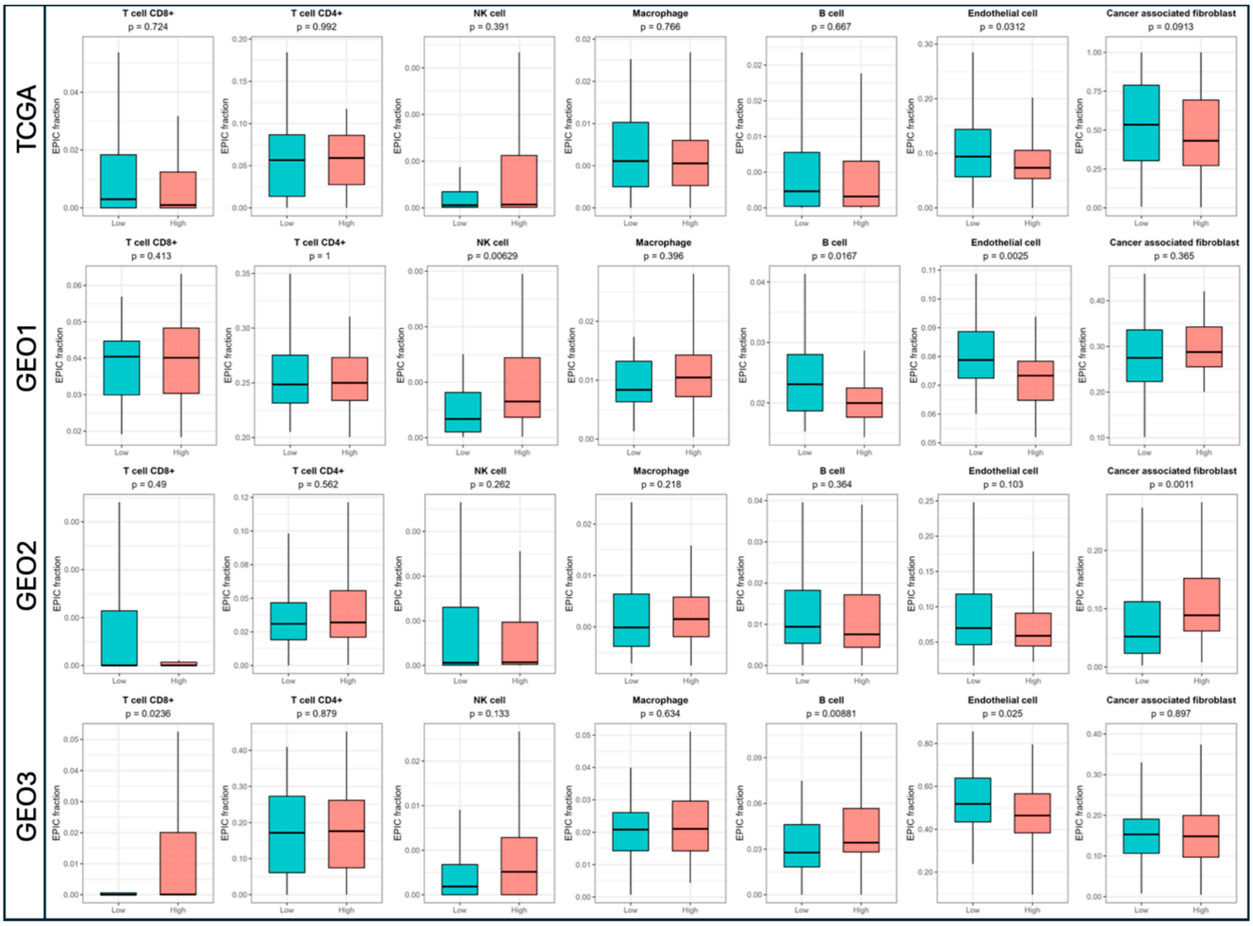

Figure S1. *Immune and stromal cell composition analysis of MKI67-high vs MKI67-low tumors.*

Box plots depicting inferred immune and stromal cell composition differences between *MKI67*-high and *MKI67*-low tumors using the EPIC cell deconvolution. Blue: MKI67-low tumors; Red: MKI67-high tumors. Statistical significance was assessed using the Mann–Whitney U test. P values are displayed above each comparison.
